# A Compact Multipartite Mitogenome of *Clausena lansium* ‘Ji Xin’ Highlights Structural Diversity Within Rutaceae

**DOI:** 10.3390/ijms27156984

**Published:** 2026-08-04

**Authors:** Changwei Bi, Zhenjun Bin, Anna Jiang, Junru Qu, Xiaoling Hao, Liyi Nong, Kaikai Meng

**Affiliations:** 1 State Key Laboratory for Tree Genetics and Breeding, Co-Innovation Center for Sustainable Forestry in Southern China, Key Laboratory of Tree Genetics and Biotechnology of Educational Department of China, Key Laboratory of Tree Genetics and Breeding of Jiangsu Province, College of Information Science and Technology & Artificial Intelligence, Nanjing Forestry University, Nanjing 210037, China; bichwei@njfu.edu.cn (C.B.); annajiang@njfu.edu.cn (A.J.); 2 Guangxi Key Laboratory of Quality and Safety Control for Subtropical Fruits, Guangxi Subtropical Crops Research Institute, Nanning 530001, China; binzj@gxaas.net (Z.B.); galaxy11q@126.com (J.Q.); hxlhaoxiaoling0423@163.com (X.H.); nongliyi11@163.com (L.N.); 3 Key Laboratory of Quality and Safety Control for Subtropical Fruit and Vegetable, Ministry of Agriculture and Rural Affairs, Guangxi Subtropical Crops Research Institute, Nanning 530001, China

**Keywords:** *Clausena lansium*, mitochondrial genome, Rutaceae, two-circle architecture, RNA editing

## Abstract

Plant mitochondrial genomes are important for respiration, organelle evolution, and phylogenetic inference, yet remain poorly characterized in *Clausena lansium*, an economically important Rutaceae fruit tree. Here, we generated the complete mitogenome of *C. lansium* cultivar ‘Ji Xin’ by integrating Oxford Nanopore and Illumina sequencing data, followed by comparative analyses of repeats, plastid-derived mitochondrial sequences, collinearity, RNA editing, and phylogenetic placement. The mitogenome consisted of two circular molecules totaling 499,621 bp, and encoded 56 genes, including 33 protein-coding genes, 20 tRNA genes, and three rRNA genes. Repeat analysis identified 206 SSRs, 21 tandem repeats, and 35 dispersed repeats, with dispersed repeats strongly enriched on mtChr1. We detected 21 plastid-derived mitochondrial fragments totaling approximately 9.23 kb, indicating limited but detectable plastid-to-mitochondrion DNA transfer. Comparative analyses revealed fragmented synteny between *C. lansium* and related Sapindales mitogenomes, suggesting lineage-specific structural rearrangement. Based on leaf rRNA-depleted lncRNA sequencing data, 402 candidate C-to-U RNA editing sites were detected in 31 mitochondrial PCGs, most of which were nonsynonymous. Phylogenetic reconstruction placed *C. lansium* within Rutaceae, close to *Limonia* and *Citrus*. These results provide a mitochondrial genomic resource for *C. lansium* and contribute to understanding mitogenome evolution in Rutaceae.

## 1. Introduction

Mitochondria play indispensable roles in eukaryotic cells, with central roles in oxidative phosphorylation, ATP production, respiratory metabolism, programmed cell death, and responses to environmental stress [1,2,3]. In plants, mitochondrial genomes (mitogenomes) provide valuable genetic resources for investigating nucleocytoplasmic interactions, organelle genome evolution, phylogenetic relationships, and cytoplasmic inheritance-related traits such as cytoplasmic male sterility [4,5,6]. Unlike the compact and relatively conserved animal mitogenomes, plant mitogenomes are highly dynamic in genome size and structural organization [7,8,9]. They often contain extensive non-coding regions, repeated sequences, plastid-derived fragments, horizontally transferred DNA, and complex structural configurations [10,11,12,13,14]. These features make plant mitogenomes an important system for investigating genome maintenance, recombination, intracellular DNA transfer, and organelle genome evolution [15,16,17,18,19].

Plant mitogenomes exhibit a striking combination of functional conservation and structural dynamism. Core protein-coding genes (PCGs) involved in oxidative phosphorylation and mitochondrial translation are generally conserved across land plants, reflecting relatively slow evolutionary rates and well-defined orthologous relationships [4,13]. Accordingly, mitochondrial PCGs have been increasingly used in plant phylogenomics and have shown potential for resolving deep phylogenetic relationships in angiosperms [20]. In contrast to this conservation at the coding-sequence level, plant mitogenomes often vary substantially in gene repertoire, intergenic content, and genome architecture among lineages. In vascular plants, this structural diversity is manifested by extensive rearrangements, weak syntenic conservation, and multiple alternative conformations, largely associated with repeat-mediated recombination [7,10,21]. Repeated sequences, particularly dispersed repeats, can promote genome isomerization and structural rearrangement and are therefore considered important drivers of plant mitogenome evolution [16,22,23,24]. In addition, plant mitochondrial transcripts often require *cis*- or *trans*-splicing of introns and extensive RNA editing, especially C-to-U editing, which can restore conserved amino acids and maintain protein function [25,26,27]. Thus, integrated analyses of gene repertoire, repeat elements, RNA editing, and phylogenetics are essential for understanding the functional and evolutionary characteristics of plant mitogenomes [28].

Despite rapid advances in organelle genomics, complete assembly of plant mitogenomes remains challenging. Plant mitochondrial DNA is difficult to isolate and enrich, and whole-genome sequencing data often contain mixed nuclear, plastid, and mitochondrial reads [14,29]. Moreover, nuclear mitochondrial DNA segments and plastid-derived mitochondrial insertions can complicate assembly, while repetitive sequences, structural heteroplasmy, and alternative mitochondrial conformations further hinder the reconstruction of complete mitogenomes [18]. Short-read sequencing approaches are often insufficient to resolve long repeats and complex graph structures [30,31], whereas long-read and HiFi sequencing technologies have substantially improved assembly contiguity and accuracy. Recent advances in plant mitogenome assembly tools and graph-based strategies [14,32,33,34,35,36], including PMAT, GSAT, HiMT, OATK, and TIPPo, have provided new opportunities to recover more complete and reliable mitogenome sequences. These developments are shifting plant mitochondrial genomics from single representative circular assemblies toward more comprehensive interpretations of dynamic and graph-like mitogenome architectures.

Within Sapindales, Rutaceae represents an informative lineage for mitogenome research, as it includes many economically important fruit, medicinal, and aromatic plants, such as *Citrus*, *Clausena*, *Phellodendron*, *Melicope*, and *Limonia*. However, Rutaceae mitogenomes remain underrepresented compared with the rapidly expanding plastid genome (plastome) resources. Recent mitogenome studies of *Citrus medica*, *Melicope pteleifolia*, and *Phellodendron amurense* have begun to reveal substantial structural diversity within Rutaceae, highlighting the need for broader taxonomic sampling in this family [37,38,39,40]. *Clausena lansium* (*C. lansium*), commonly known as wampee, is a Rutaceae fruit tree cultivated in southern China and other subtropical regions [41]. It is valued for both edible fruits and bioactive metabolites, particularly carbazole alkaloids [41,42]. Although plastome and nuclear genome resources have been reported for *C. lansium*, improving understanding of its taxonomy, evolution, and specialized metabolism [41,43], its mitogenome remains insufficiently characterized.

In this study, we assembled and characterized the complete mitogenome of *C. lansium* cultivar ‘Ji Xin’ and performed comprehensive comparative genomic analyses. We characterized its genome architecture, gene repertoire, repeat elements, plastid-derived mitochondrial sequences, and RNA editing events. Comparative analyses with related species were further performed to evaluate collinearity and structural variation, and the phylogenetic placement of *C. lansium* was reconstructed using conserved mitochondrial genes. This work expands mitochondrial genomic resources for Rutaceae and offers new insights into comparative studies of mitogenome evolution in Sapindales.

## 2. Results

### 2.1. General Characteristics of the C. lansium Mitogenome

**Figure 1:**
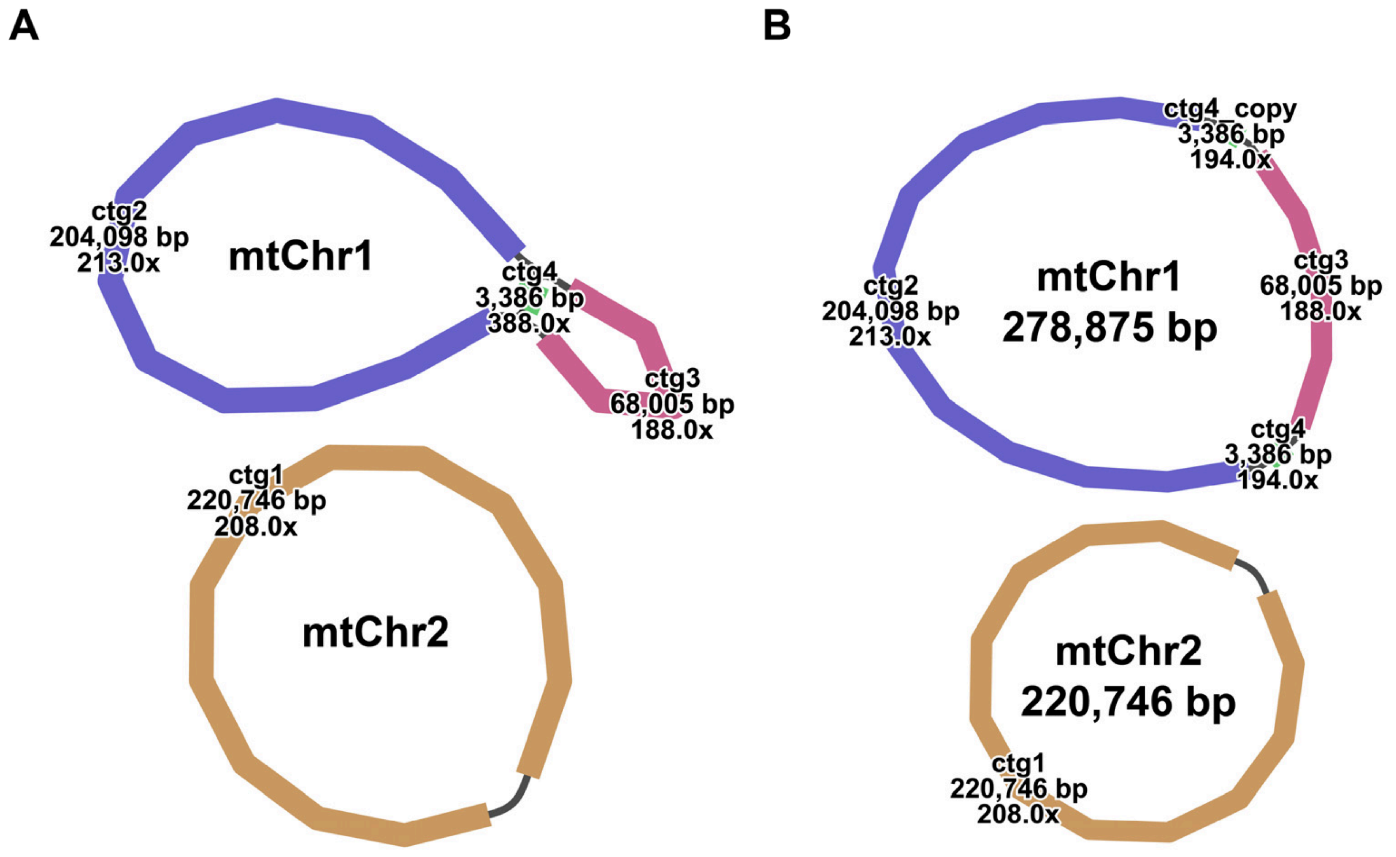
Assembly graph of the *Clausena lansium* mitogenome before (**A**) and after (**B**) graph resolution.

The mitogenome of *C. lansium* cultivar ‘Ji Xin’ was resolved into two circular molecules, designated mtChr1 and mtChr2 (Figure 1). The complete mitogenome was 499,621 bp in length, with mtChr1 and mtChr2 accounting for 278,875 bp and 220,746 bp, respectively. The overall GC content was 45.03%, with comparable values for mtChr1 (45.27%) and mtChr2 (44.73%). The two circular mitogenome sequences have been submitted to GenBank under accession numbers PZ533874.1 and PZ533875.1 (Table S1). A total of 56 genes were identified, comprising 33 PCGs, 20 tRNA genes, and three rRNA genes (Table 1 and Table S2). Among these genes, only two tRNA genes, *trnP*-UGG and *trnS*-UGA, were present in multiple copies. The gene regions spanned 36,689 bp, representing 8.59% of the complete mitogenome. Of these, PCGs accounted for 29,967 bp, whereas tRNA and rRNA genes accounted for 1539 bp and 5183 bp, respectively. Gene distribution differed between the two mitochondrial chromosomes: mtChr1 harbored 33 genes, including 17 PCGs, 13 tRNA genes, and all three rRNA genes, whereas mtChr2 contained 23 genes, including 16 PCGs and seven tRNA genes.

**Figure 2:**
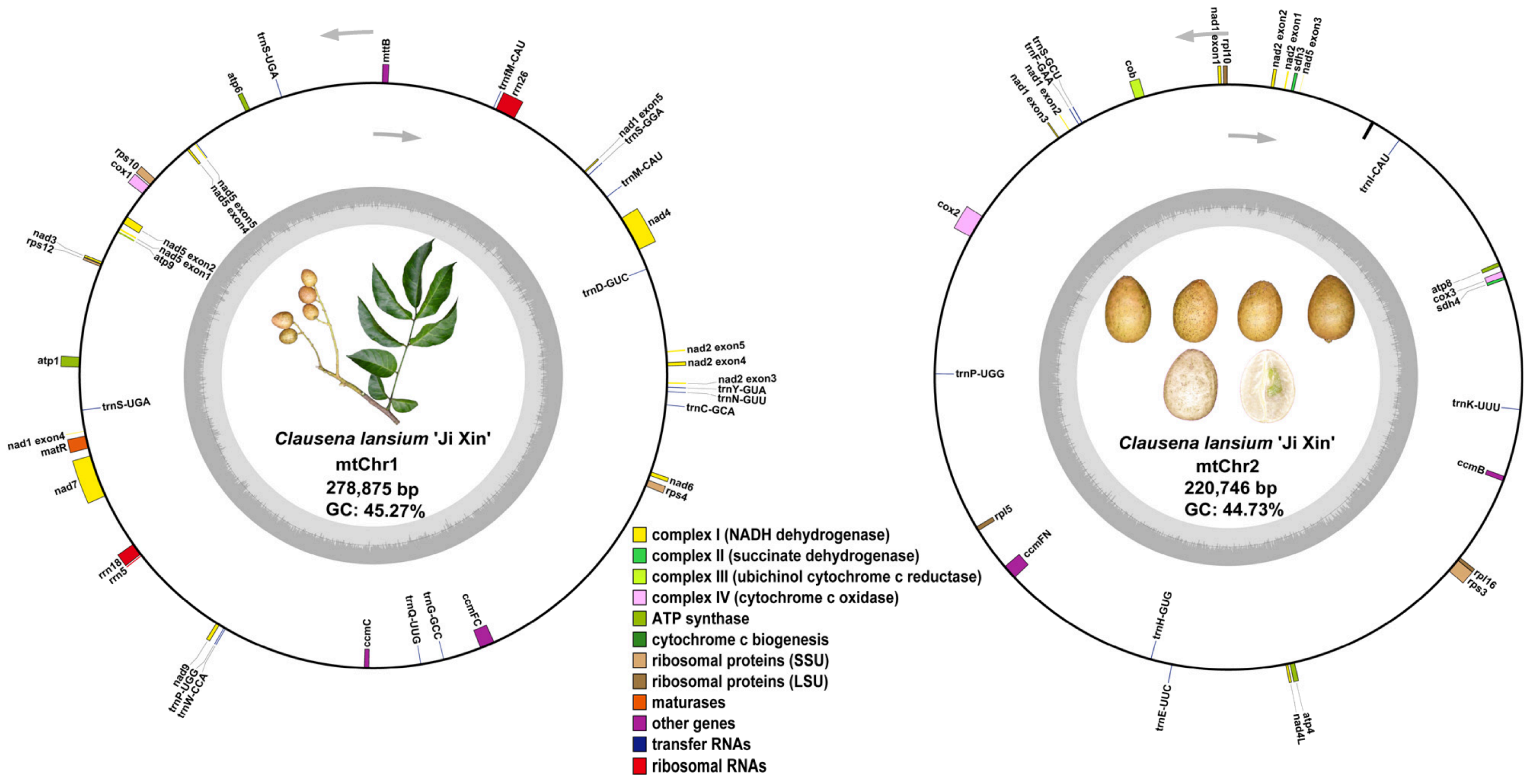
Genome graph of the two circular mitochondrial chromosomes of *Clausena lansium*: (**left**) mtChr1; (**right**) mtChr2. Genes transcribed clockwise and counter-clockwise are shown on the inside and outside of the circles, respectively. Genes are color-coded according to their functional categories. The dark gray histogram on the inner circle represents GC content.

Twenty-one *cis*-spliced introns were identified in eight PCGs, with several NADH dehydrogenase genes (*nad1*, *nad2*, *nad4*, *nad5*, and *nad7*) containing multiple introns (Figure 2 and Table S2). These intron-containing genes were entirely confined to one mitochondrial chromosome, whereas *nad1*, *nad2*, and *nad5* extended both chromosomes. Start and stop codon usage was generally conserved among the 33 PCGs. Most PCGs initiated with the canonical ATG codon, while *nad1*, *nad4L*, and *rps10* carried ACG as the genomic start codon, which may be edited to AUG at the transcript level (Table S2). The start codon of *rpl16* remained unresolved. Among the identified stop codons, TAA, TGA, and TAG occurred in 14, 10, and six genes, respectively. In contrast, *ccmFC*, *atp9*, and *rps10* harbored CGA as the genomic terminal codon, which may be converted to UGA in the transcript through C-to-U RNA editing.

### 2.2. Repeat Elements in the C. lansium Mitogenome

**Figure 3:**
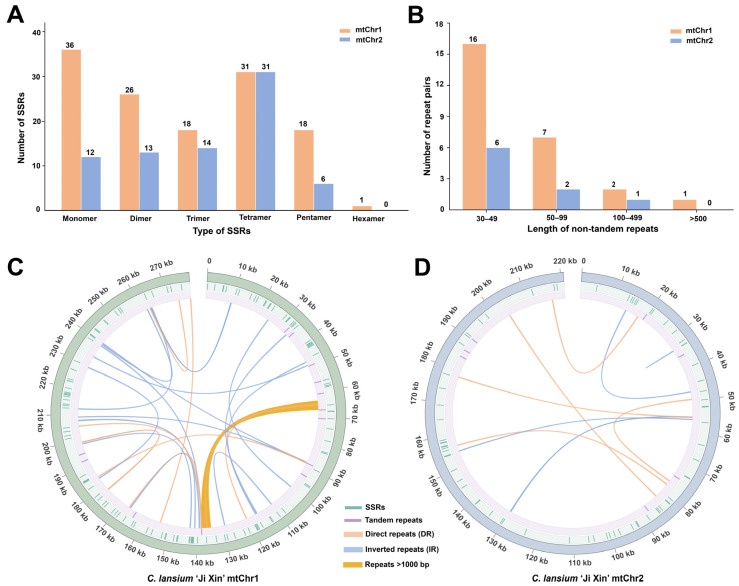
Characterization of repetitive sequences in the two mitochondrial chromosomes of *Clausena lansium*: (**A**) Frequency of SSRs. (**B**) Frequency of dispersed repeats. (**C**,**D**) Circular maps showing the distribution of repeat elements in mtChr1 (**C**) and mtChr2 (**D**). Direct repeats and inverted repeats are connected by orange and blue links, respectively, while repeats longer than 1 kb are highlighted in orange.

The *C. lansium* mitogenome contained 206 simple sequence repeats (SSRs), including 130 loci on mtChr1 and 76 loci on mtChr2 (Figure 3A). These SSRs were broadly and relatively evenly distributed across the two mitochondrial chromosomes, with no obvious large SSR-depleted regions. Based on repeat-unit length, the SSRs comprised 48 mono-, 39 di-, 32 tri-, 62 tetra-, 24 penta-, and one hexanucleotide repeat (Table S3). In addition, 21 tandem repeats were detected, with 14 located on mtChr1 and seven on mtChr2 (Table S4). These tandem repeats varied from 32 to 90 bp in length, with unit sizes ranging from 14 to 36 bp and copy numbers from 2.0 to 3.1. Most tandem repeats showed high sequence similarity, and four displayed complete sequence identity. The longest tandem repeat, TR20, was located on mtChr2 and extended from 186,280 to 186,369 bp, with a 36 bp repeat unit and 90% sequence similarity.

The *C. lansium* mitogenome contained 35 pairs of dispersed repeats (Table S5; Figure 3B), with a cumulative length of 11,019 bp, representing 2.21% of the total mitogenome (Table 1). These repeats showed a strong bias toward mtChr1, which contained 26 repeat pairs totaling 10,010 bp, whereas mtChr2 contained only nine repeat pairs totaling 1009 bp. Dispersed repeats were dominated by short repeat pairs, with 31 pairs shorter than 100 bp and accounting for 88.5% of the total, while most repeats occurred in two copies. Notably, the only repeat longer than 1 kb was a long direct repeat located on mtChr1. Additionally, using stricter criteria for inter-molecular repeat detection, 12 shared repeats were identified between mtChr1 and mtChr2 (Table S6), ranging from 56 to 269 bp in length, including nine direct repeats and three inverted repeats.

Overall, the repeat profile of the *C. lansium* mitogenome was characterized by abundant short SSRs, unevenly distributed tandem repeats, and an mtChr1-enriched set of dispersed repeats (Figure 3C,D), which may provide potential substrates for mitogenome recombination and structural variation.

### 2.3. Plastid-Derived Mitochondrial Sequences in the C. lansium Mitogenome

**Figure 4:**
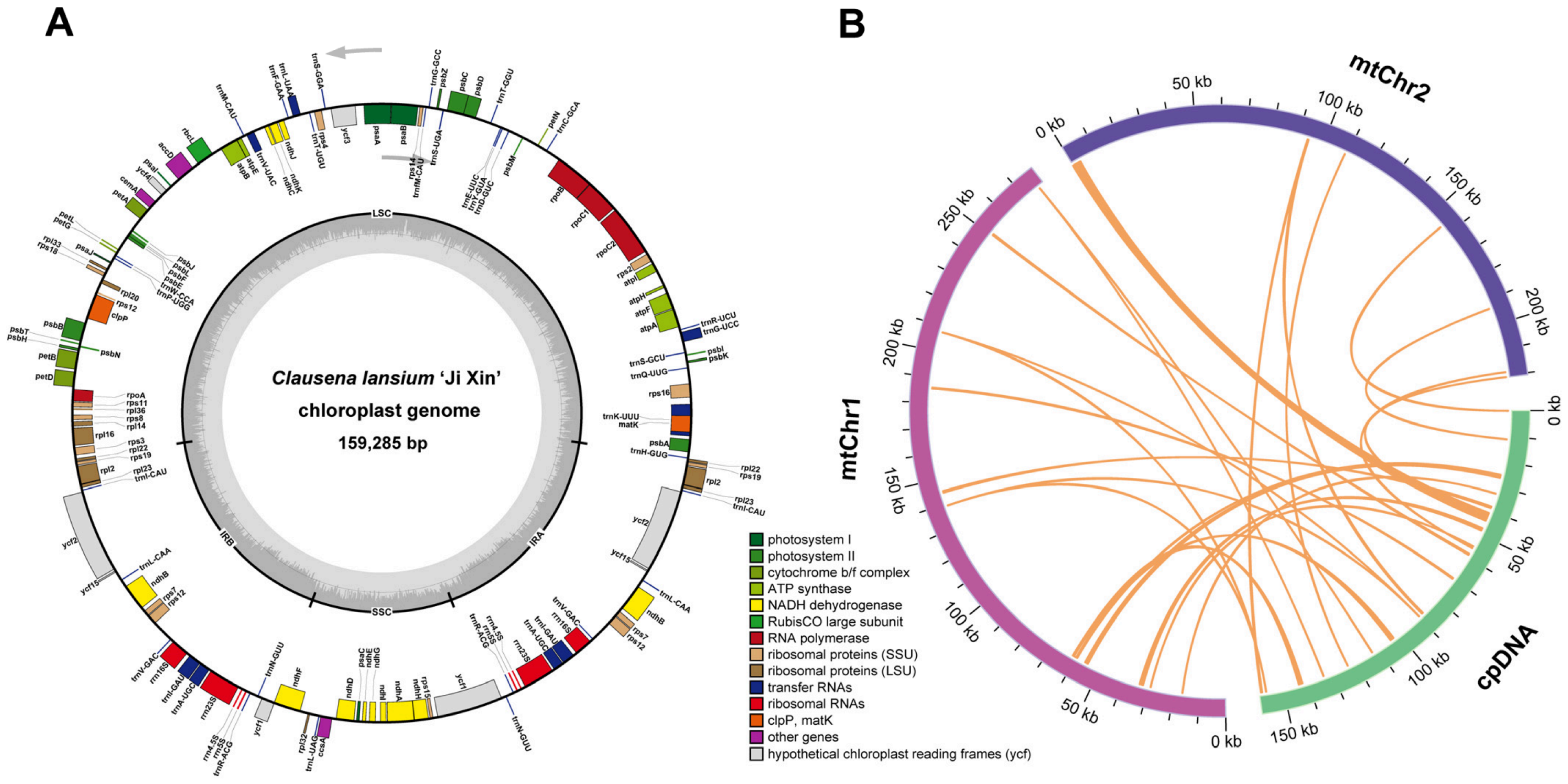
Plastid genome map and mitochondrial plastid DNA transfers (MTPTs) in *Clausena lansium*: (**A**) Gene map of the *C. lansium* plastid genome. (**B**) Homologous fragments between the plastid genome and the two mitochondrial chromosomes (mtChr1 and mtChr2). Orange ribbons indicate MTPTs.

Alignment between the plastid and mitochondrial genomes revealed 21 plastid-derived mitochondrial sequences (MTPTs), totaling approximately 9.23 kb and representing 1.85% of the *C. lansium* mitogenome (Figure 4 and Table S7). Fourteen MTPT fragments were located on mtChr1, collectively accounting for 5285 bp, whereas seven fragments were located on mtChr2 and accounted for 3942 bp. Most transferred fragments were short; however, one 3236 bp fragment on mtChr2 showed 99.94% sequence identity to the plastome and contained partial *psaB* and *psaA* sequences. MTPT-associated annotations included partial plastid protein-coding genes, such as *rpoB*, *psbC*, *rbcL*, *ycf3*, *psaA*, *psaB*, *ndhI*, *atpA*, *atpB*, *atpE*, and *rpl2*, as well as several plastid-derived tRNA genes. Complete or partial tRNA gene annotations within MTPTs included *trnS*-UGA, *trnD*-GUC, *trnH*-GUG, *trnS*-GGA, *trnI*-CAU, *trnM*-CAU, *trnW*-CCA, *trnP*-UGG, and *trnN*-GUU (Table S7). These results indicate that plastid-to-mitochondrion sequence transfer contributed a detectable but limited fraction of the *C. lansium* mitogenome.

### 2.4. Comparative Mitogenome Collinearity and Structural Divergence

**Figure 5:**
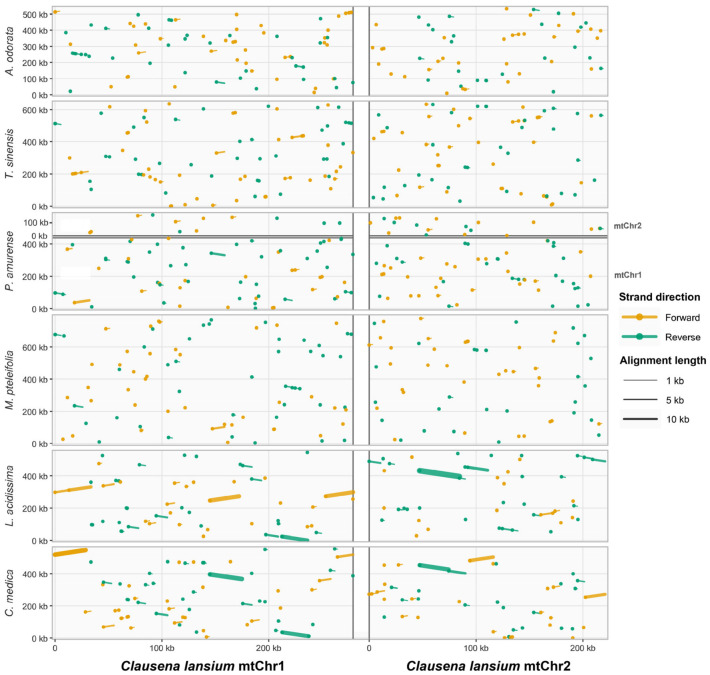
Mitogenome collinearity between the two mitochondrial chromosomes of *Clausena lansium* and six closely related species. Orange and green segments indicate alignments in the forward (+) and reverse (−) orientations, respectively, and line thickness is proportional to the alignment length.

Pairwise collinearity analyses were performed using the *C. lansium* mitogenome as the reference and six related angiosperm mitogenomes as query genomes, including *C. medica*, *Limonia acidissima*, *M. pteleifolia*, *P. amurense*, *Toona sinensis*, and *Aglaia odorata* (Figure 5 and Table S8). The highest proportions of the *C. lansium* mitogenome covered by non-redundant syntenic blocks were observed in comparisons with *L. acidissima* (80.42%) and *C. medica* (79.32%). These two comparisons also exhibited the longest average syntenic block lengths, at 4.00 kb and 3.96 kb, respectively, with corresponding non-redundant query coverages of 73.77% and 74.56%. In contrast, comparisons with *M. pteleifolia*, *P. amurense*, *T. sinensis*, and *A. odorata* produced more fragmented collinearity patterns, characterized by a greater number of shorter syntenic blocks and markedly lower mitogenome coverage. For example, *P. amurense* contained 153 collinearity blocks but showed only 39.23% non-redundant reference coverage and 34.76% query coverage. Comparisons with *T. sinensis* and *A. odorata* showed reference coverages of 34.00% and 31.57%, respectively. The comparison with *M. pteleifolia* identified 144 relatively short syntenic blocks, with a mean length of 1.02 kb, covering 29.36% of the *C. lansium* reference genome and 18.89% of the *M. pteleifolia* query genome. Together, these results indicate extensive structural divergence among Sapindales mitogenomes, even among relatively closely related lineages.

### 2.5. RNA Editing Events in the C. lansium Mitogenome

**Figure 6:**
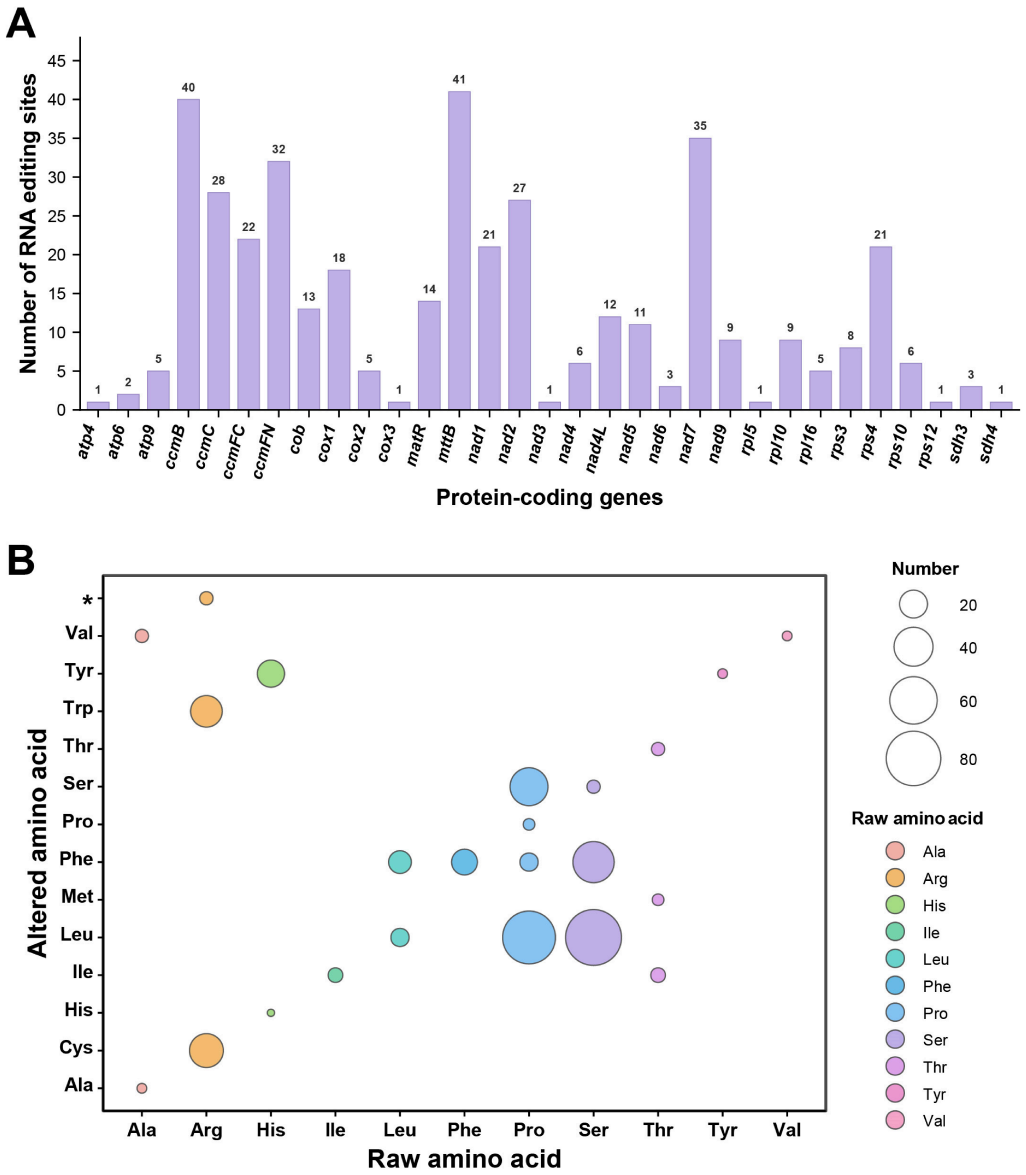
Candidate RNA editing sites detected in the *Clausena lansium* mitogenome using leaf lncRNA-seq data. (**A**) Distribution of candidate RNA editing sites across mitochondrial PCGs. (**B**) Amino acid conversions caused by candidate RNA editing events. The asterisk on the vertical axis represents stop codons. Bubble size indicates the frequency of each amino acid conversion.

Using leaf rRNA-depleted lncRNA-seq data, we detected 402 candidate C-to-U RNA editing sites unevenly distributed across 31 of the 33 PCGs in *C. lansium* (Figure 6 and Table S9). The highest numbers of editing sites were detected in *mttB* (41 sites), *ccmB* (40), *nad7* (35), *ccmFN* (32), *ccmC* (28), and *nad2* (27), whereas several genes contained only one or a few predicted sites (Figure 6A). At the codon level, editing events were predominantly concentrated at the second codon position (211 sites), followed by the first (146 sites) and third positions (45 sites). The estimated editing levels ranged from 0.1 to 1, with a mean value of 0.793, indicating that many candidate sites were supported by relatively high editing levels. Among the 402 candidate sites, 354 were nonsynonymous, whereas 48 were synonymous or did not alter the encoded amino acid. The most frequent amino acid conversions were Ser-to-Leu, Pro-to-Leu, Ser-to-Phe, Pro-to-Ser, Arg-to-Cys, and Arg-to-Trp (Figure 6B). Overall, the editing profile was dominated by conversions from hydrophilic or structurally constrained residues, particularly serine, proline, and arginine, to more conserved amino acid states, such as leucine, phenylalanine, cysteine, and tryptophan. These patterns are consistent with the established role of C-to-U RNA editing in restoring evolutionarily conserved amino acids and maintaining functional mitochondrial proteins in plants.

### 2.6. Phylogenetic Analysis of C. lansium Based on Mitochondrial PCGs

**Figure 7:**
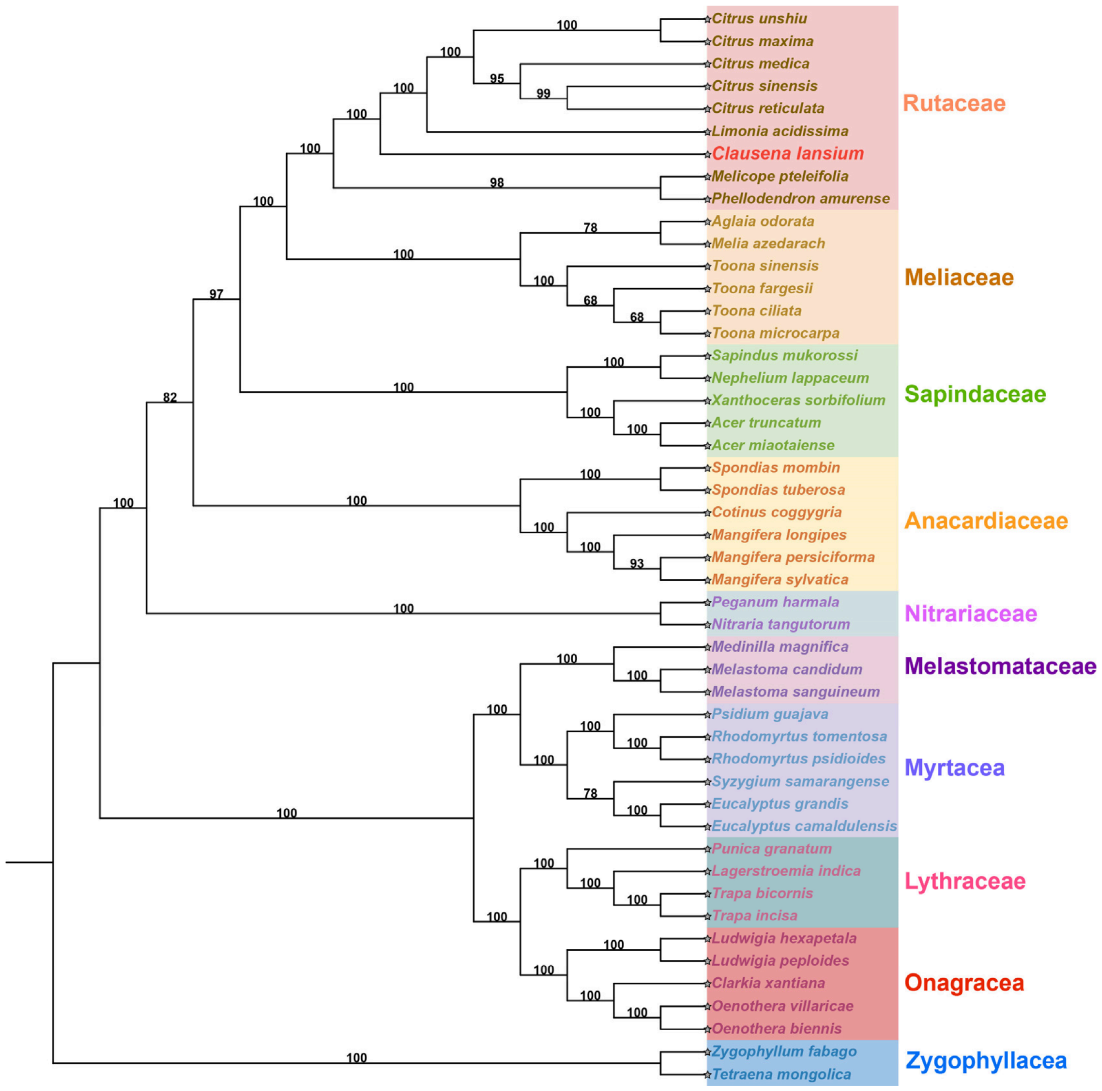
Maximum-likelihood phylogenetic tree inferred from conserved mitochondrial PCGs of representative angiosperms. Bootstrap support values are shown at the nodes. *Clausena lansium* is highlighted in red.

Phylogenetic relationships were inferred using a maximum-likelihood approach based on conserved mitochondrial PCGs from the sampled angiosperms, representing ten families: Rutaceae, Meliaceae, Sapindaceae, Anacardiaceae, Nitrariaceae, Melastomataceae, Myrtaceae, Lythraceae, Onagraceae, and Zygophyllaceae (Figure 7 and Table S1). The recovered tree well-supported family-level clades, indicating that the mitochondrial PCG dataset provided sufficient phylogenetic signal to resolve broad relationships among the sampled taxa. *C. lansium* was placed within Rutaceae with strong bootstrap support and was closely associated with the *Limonia* and *Citrus* lineages. In contrast, *M. pteleifolia* and *P. amurense* formed a neighboring branch within Rutaceae. This topology is consistent with the taxonomic placement of *C. lansium* in Rutaceae and provides a mitochondrial genomic framework for comparative analyses of mitogenome evolution within Sapindales.

## 3. Discussion

The *C. lansium* mitogenome is 499,621 bp in length and is represented by two circular molecules, indicating a relatively compact and structurally distinctive architecture among the Rutaceae mitogenomes examined in this study. As shown in Table S1, the ten other Rutaceae mitogenomes range in size from 517,373 bp in *Tetradium ruticarpum* to 780,107 bp in *M. pteleifolia*, with an average size of approximately 580.8 kb. Thus, the *C. lansium* mitogenome is smaller than all the other Rutaceae mitogenomes included in this comparison and is also more compact than the mitogenomes used for collinearity analysis. Most Rutaceae mitogenomes in the comparison set are represented as single circular molecules, whereas *P. amurense*, *T. ruticarpum*, and *C. lansium* have been reported as multipartite circular assemblies [39]. Therefore, the two-molecule representation of the *C. lansium* mitogenome is uncommon but not unique within the family. Given the dynamic and recombinational nature of plant mitogenomes, this architecture should be regarded as a high-confidence reference configuration rather than evidence that only two stable molecules exist *in vivo*. Broader sampling across *Clausena* and other Rutaceae lineages will be required to determine whether this architecture is cultivar-specific, species-specific, or more widely distributed within the family.

Plant mitogenome assembly is intrinsically challenging because mitochondrial DNA is often present at low abundance in total genomic DNA, co-occurs with plastid and nuclear organellar fragments, and contains repeats that can generate branching paths in assembly graphs. These features make it difficult to distinguish true multipartite mitochondrial molecules from alternative graph resolutions or repeat-mediated isoforms. Moreover, plant mitogenomes are highly dynamic and may exist *in vivo* as complex populations of alternative molecular conformations rather than as a single invariant chromosome [7,44]. Accordingly, published assemblies often represent the dominant graph-resolved configuration supported by the available sequencing data. As emphasized by Ni et al. [29], plant mitogenome assembly can be complicated by branched structures and repeat-mediated recombination. Therefore, the two circular molecules of the *C. lansium* mitogenome should be regarded as a high-confidence reference representation rather than a definitive description of all possible *in vivo* conformations. Consistent with this interpretation, we detected 12 shared repeats between mtChr1 and mtChr2, including nine direct and three inverted repeats of 56–269 bp, suggesting that inter-molecular homologous recombination may potentially generate alternative subgenomic configurations. Future read-depth analyses, long-read validation across junctions, and recombination assays will be useful for determining whether additional subgenomic or alternative conformations are present.

Repeat sequences are important drivers of plant mitogenome evolution because they provide substrates for homologous recombination and structural rearrangement [10]. The repeat landscape of the *C. lansium* mitogenome is relatively modest compared with those of several reported Rutaceae mitogenomes. In this study, 206 SSRs, 21 tandem repeats, and only 35 dispersed repeat pairs were identified. By contrast, *C. medica* contains 633 repeats, including 386 dispersed repeats, with long inverted repeats exceeding 11 kb proposed to facilitate recombination [37]. *M. pteleifolia* contains 507 repetitive sequences, including 235 dispersed repeats [38], whereas *P. amurense* contains 310 dispersed repeats, with repeat-mediated recombination inferred in its mitogenome [39]. Compared with these Rutaceae references, the *C. lansium* mitogenome is therefore characterized by a lower dispersed-repeat burden, consistent with its relatively compact genome size. This pattern is relevant because dispersed repeats are major substrates for plant mitogenome recombination. Wynn and Christensen showed that recombination potential is strongly size dependent: large repeats can recombine constitutively and generate alternative genomic conformations, whereas short repeats are usually less active under normal conditions [10]. In the *C. lansium* mitogenome, most dispersed repeats were shorter than 100 bp (31 pairs, 88.5%), and only one repeat exceeded 1 kb, namely a 3386 bp direct repeat on mtChr1. Although the relatively low repeat content of the *C. lansium* mitogenome may indicate lower overall recombination potential, its fragmented collinearity with related Sapindales species still reflects substantial structural divergence. This pattern was particularly pronounced in the comparison with *M. pteleifolia*, whose substantially larger and more repeat-rich mitogenome may have experienced greater repeat-mediated recombination and lineage-specific gain or loss of intergenic sequences [4,10,38].

Plastid-to-mitochondrion intracellular gene transfer is widespread in angiosperms and represents an important source of non-native mitochondrial DNA [15,45]. In *C. lansium*, 21 MTPTs totaling approximately 9.23 kb accounted for 1.85% of the mitogenome. By comparison, *L. acidissima*, a closely related Rutaceae species, contains 11 MTPTs totaling 25,167 bp, equivalent to approximately 4.62% of its mitogenome, with the longest fragment reaching 8477 bp [45]. Thus, despite having more detected MTPTs, *C. lansium* contains a substantially lower total amount of plastid-derived DNA, suggesting greater fragmentation or reduced retention of transferred sequences. However, differences in detection thresholds among studies may also affect MTPT counts. The MTPT proportion in *C. lansium* was also lower than the 4.07% reported for *M. pteleifolia* [38]. Recent comparative analyses suggest that MTPT diversity may arise from the transfer of large plastid DNA tracts followed by lineage-specific deletion, duplication, substitution, and rearrangement [45]. Accordingly, the numerous short MTPTs and partial plastid genes in *C. lansium* may reflect post-integration erosion, whereas its 3236 bp fragment with 99.94% identity may represent a relatively recent or highly conserved transfer. Although most partial protein-coding sequences are unlikely to remain functional, plastid-derived tRNAs and other retained fragments may provide genetic material for recombination and structural evolution.

Conserved mitochondrial PCGs provide useful phylogenetic signal for resolving broad angiosperm relationships because mitochondrial coding sequences generally evolve more slowly than plastid or nuclear sequences. Consistent with recent angiosperm-scale analyses based on 41 mitochondrial PCGs from 481 angiosperm species [20], our maximum-likelihood phylogeny recovered well-supported family-level clades across the sampled taxa and placed *C. lansium* firmly within Rutaceae. Within this family, *C. lansium* was positioned close to the *Limonia* and *Citrus* lineages, whereas *M. pteleifolia* and *P. amurense* formed a neighboring branch. This topology supports the accepted taxonomic placement of *Clausena* and highlights the utility of mitochondrial PCGs for phylogenetic inference within Sapindales. Rutaceae mitogenomic resources remain limited, with most sampled genera represented by only a single species and many genera lacking complete mitogenome data. Broader sampling across *Clausena*, *Limonia*, and other Rutaceae lineages will therefore be necessary to further evaluate the stability of these phylogenetic relationships. However, phylogenetic proximity should not be equated with mitogenome structural conservation. Collinearity analysis revealed fragmented synteny between *C. lansium* and several Sapindales mitogenomes, including those of closely related Rutaceae species. This contrast reflects a common feature of plant mitochondrial evolution: conserved coding regions can retain phylogenetic signal, whereas genome architecture may be rapidly reshaped by repeat-mediated recombination, MTPTs, and lineage-specific rearrangements [13,19,45]. Thus, this study provides both a mitochondrial phylogenetic reference for an underrepresented Rutaceae genus and evidence that sequence conservation and structural diversification are decoupled within Sapindales.

## 4. Materials and Methods

### 4.1. Plant Material, DNA Extraction and Sequencing

Fresh leaves were sampled from *C. lansium* cultivar ‘Ji Xin’ grown at the Subtropical Botanical Garden of the Guangxi Institute of Tropical Crops in Nanning, Guangxi, China. The collected leaves were immediately frozen in liquid nitrogen and preserved at −80 °C prior to DNA isolation. Total genomic DNA was isolated using a modified CTAB method. DNA quality was assessed by agarose gel electrophoresis, while DNA quantity and purity were determined with a NanoDrop 2000 spectrophotometer (Thermo Fisher Scientific, Waltham, MA, USA). Qualified DNA was subsequently split into two aliquots for short- and long-read library construction. The short-read library was sequenced on the Illumina NovaSeq 6000 platform (Illumina, San Diego, CA, USA), whereas the long-read library was generated using the Oxford Nanopore Technologies PromethION platform (Oxford Nanopore Technologies, Oxford, UK). The resulting Illumina and ONT datasets were combined for mitochondrial genome assembly.

### 4.2. Mitogenome Assembly and Annotation

A long-read-first strategy, followed by mitochondrial read enrichment and hybrid assembly, was used to generate the *C. lansium* mitogenome. ONT long reads were initially assembled *de novo* using Flye v2.9.3 with default parameters [46], generating FASTA contigs and a GFA assembly graph. All the assembled contigs were then used to build a local nucleotide database using makeblastdb. The *C. medica* mitogenome (GenBank accession PQ636878.1) was then used as a query against this database using BLASTn v2.13.0 [47] with the parameters ‘-evalue 1 × 10^−5^ -outfmt 6 -max_hsps 10 -word_size 7’. Candidate mitochondrial contigs were identified according to sequence similarity and further inspected in the assembly graph using Bandage v0.8.1 [48], yielding an initial mitochondrial assembly. ONT and Illumina reads were independently mapped back to these mitochondrial contigs using minimap2 v2.26 [49], and the aligned reads were extracted to generate mitochondrial-enriched datasets. These long- and short-read datasets were subsequently subjected to hybrid assembly using Unicycler v0.5.1 [50]. The final assembly graph was manually inspected in Bandage v0.8.1, from which the *C. lansium* mitogenome was represented as two circular molecules.

The *C. lansium* mitogenome was first annotated using the PMGA online platform [51] with a reference dataset of 319 angiosperm mitogenomes. Putative tRNA and rRNA genes were further verified using tRNAscan-SE v2.0.12 [52] and BLASTn v2.13.0 [47], respectively. Resulting annotations were manually inspected and refined using MacVector v18.8 to resolve potential errors in gene boundaries and feature assignments. Finally, graphical maps of the *C. lansium* mitogenome were generated using the online tool PMGmap [53].

### 4.3. Identification of Repeat Elements

Following recent repeat-analysis workflows for Rutaceae mitogenomes [37,38,43], the two mitochondrial molecules of *C. lansium* were analyzed separately. Simple sequence repeats were detected using MISA v2.1 [54] with motif lengths of one to six nucleotides and minimum repeat numbers of 10, 5, 4, 3, 3, and 3, respectively. Tandem repeats were predicted using Tandem Repeats Finder v4.09 [55] with the parameter settings 2, 7, 7, 80, 10, 50, and 500. Dispersed repeats were detected using the Python script ROUSFinder2.0.py (https://github.com/flydoc2000/ROUSfinder, accessed on 31 May 2026) [10], with the sequence identity threshold and minimum repeat length set to 90% and 30 bp, respectively. To assess potential recombination between mtChr1 and mtChr2 mediated by shared homologous sequences, inter-molecular repeats were further searched using stricter thresholds of 98% identity and 50 bp minimum length. The mitogenomic distributions of these identified repeats were illustrated using Circos v0.69.9 [56].

### 4.4. Detection of Plastid-Derived Mitochondrial Sequences

The *C. lansium* plastome used for MTPT analysis was *de novo* assembled from Illumina sequencing reads generated from the same ‘Ji Xin’ individual using GetOrganelle v1.7.7 [31]. The assembled plastome was first annotated with CPGAVAS2 [57], and the resulting annotation was subsequently inspected and refined using CPGView [58] to ensure accurate gene boundaries and feature assignments. The plastome was then aligned against the two mitochondrial molecules of *C. lansium* using BLASTn v2.13.0 with the following parameters: -word_size 9 -evalue 1 × 10^−5^ -reward 2 -gapopen 5 -gapextend 2 -penalty -3 -outfmt 6. Homologous regions showing an alignment length ≥ 30 bp and sequence identity ≥ 80% were considered putative MTPTs. Plastid genes overlapping these transferred fragments were identified based on the plastome annotation. Finally, the genomic distribution and correspondence of MTPTs between the plastome and mitogenome were visualized using Circos v0.69.9 [56].

### 4.5. Mitogenome Collinearity Analysis

To assess structural conservation and rearrangement patterns in the *C. lansium* mitogenome, mitogenomes from six closely related representative species were obtained from the NCBI Nucleotide database, including *C. medica*, *M. pteleifolia*, *P. amurense*, *L. acidissima*, *T. sinensis*, and *A. odorata* (Table S1). Pairwise whole-genome alignments were performed by aligning each mitogenome against *C. lansium* using the nucmer program implemented in MUMmer v4 [59]. The resulting delta files were filtered using delta-filter to retain high-confidence collinear blocks, with thresholds > 80% sequence identity and >100 bp alignment length. The filtered alignments were then parsed using show-coords to obtain the genomic coordinates, alignment lengths, and sequence identities of homologous regions. Finally, collinearity relationships between the *C. lansium* mitogenome and the six comparative mitogenomes were visualized using an in-house R script.

### 4.6. Analysis of RNA Editing Events

Total RNA isolated from *C. lansium* leaves was used to construct an rRNA-depleted library for lncRNA sequencing. The library was prepared without poly(A) selection and therefore retained both coding and non-coding long transcripts, including mitochondrial mRNAs. LncRNA sequencing data from *C. lansium* leaves were used to identify mitochondrial RNA-editing events. High-quality RNA-seq reads were aligned to the mitochondrial PCGs using BWA-MEM [60] with the parameters ‘-t 20 -B 4 -O 6 -E 1’. To minimize read loss at CDS boundaries, each PCG sequence included the full coding region together with 100 bp upstream and downstream flanking sequences. The resulting alignments were filtered using sambamba [61] to remove unmapped, secondary, and supplementary reads, retaining only alignments with a mapping quality ≥ 20. Nucleotide composition at each mapped position was then examined using REDItools v2 [62]. Candidate RNA-editing sites were defined as reference cytosine positions supported by a predominant thymine signal in the RNA reads. Only sites with an editing frequency > 0.1 were retained. Sites located in the artificial flanking regions were excluded, and final coordinates were adjusted to correspond to their actual positions within the CDS.

### 4.7. Phylogenetic Analysis

To clarify the phylogenetic position of *C. lansium* within Sapindales, mitogenome sequences from closely related representative angiosperm taxa were obtained from the NCBI Nucleotide database. The sampling comprised ten families (Table S1), including Rutaceae (nine species), Meliaceae (six species), Sapindaceae (five species), Anacardiaceae (six species), Nitrariaceae (two species), Melastomataceae (three species), Myrtaceae (six species), Lythraceae (four species), Onagraceae (five species), and Zygophyllaceae (two species). Shared mitochondrial gene alignments were generated with MAFFT v7.505 [63] and subsequently concatenated into a combined matrix for phylogenetic reconstruction. A maximum-likelihood tree was inferred using IQ-TREE v1.6.12 [64] with 1000 ultrafast bootstrap replicates under the parameters ‘--alrt 1000 -B 1000’. The resulting tree was visualized and annotated using iTOL v6 [65], with taxa grouped and colored according to family-level classification.

## Figures and Tables

**Table 1:** Genomic features of the *Clausena lansium* mitogenome.

Statistical Information	Total Mitogenome	mtChr1	mtChr2
Accession number	PZ533874-75	PZ533874	PZ533875
Length (bp)	499,621	278,875	220,746
GC content (%)	45.03	45.27	44.73
Number of genes	56	33	23
Number of PCGs	33	17	16
Number of rRNA genes	3	3	0
Number of tRNA genes	20	13	7
Total length of genes (bp)	36,689 (8.59%)	23,220 (8.33%)	13,469 (6.10%)
Total length of PCGs (bp)	29,967	17,028	12,939
Total length of tRNAs (bp)	1539	1009	530
Total length of rRNAs (bp)	5183	5183	0
Number of *cis*-spliced introns	21	14	7
Total repeat length (bp)	11,019 (2.21%)	10,010 (3.59%)	1009 (0.46%)
Number of repeat pairs	35	26	9
Average repeat size (bp)	156.17	191.65	53.67
Average copy number	2.06	2.04	2.11

## Data Availability

The data supporting this study have been deposited in NCBI GenBank under the following accession numbers: PZ547818.1 (plastome) and PZ533874.1-PZ533875.1 (mitogenome). The Illumina and ONT raw sequencing data have been deposited under SRA accession numbers SRR39458669 and SRR39458670, respectively.

## Supplementary Materials

The following supporting information can be downloaded at https://www.mdpi.com/article/10.3390/ijms27156984/s1.
